# Nano-sized zinc addition enhanced mammary zinc translocation without altering health status of dairy cows

**DOI:** 10.1016/j.aninu.2021.06.003

**Published:** 2021-09-04

**Authors:** Jie Cai, Chao Miao, Yi Chen, Yunyi Xie, Jianxin Liu, Diming Wang

**Affiliations:** Key Laboratory of Molecular Animal Nutrition of Ministry of Agriculture, College of Animal Sciences, Institute of Dairy Science, Zhejiang University, Hangzhou 310058, China

**Keywords:** Zinc, Nano-size, Oxidative stress, Lactation performance, Mammary gland permeability

## Abstract

This study aimed to evaluate role of nano-sized zinc (Zn) on lactation performance, health status, and mammary permeability of lactating dairy cows. Thirty multiparous dairy cows with similar days in milk (158 ± 43.2) and body weight (694 ± 60.5 kg) were chosen based on parity and milk production and were randomly assigned to 3 treatment groups: basal diet (control, 69.6 mg/kg of Zn adequate in Zn requirement), basal diet additional Zn-methionine (Zn-Met, providing 40 mg/kg of Zn), and basal diet additional nano-sized Zn oxide (nZnO, providing 40 mg/kg of Zn). The study lasted for 10 wk, with the first 2 wk as adaptation. Feed intake, milk yield and the related variables, and plasma variables were determined every other week. Blood hematological profiles were determined in the 8th week of the study. We found that feed intake, milk yield, and milk composition were similar across the 3 groups. The nZnO- and Zn-Met-fed cows had greater milk Zn concentrations in the milk (3.89 mg/L (Zn-Met) and 3.93 mg/L (nZnO)) and plasma (1.25 mg/L (Zn-Met) and 1.29 mg/L (nZnO)) than the control cows (3.79 mg/L in milk and 1.21 mg/L in plasma). The nZnO-fed cows had higher Zn concentrations in plasma but not in milk compared to Zn-Met-fed cows. The Zn appearance in milk was greater in nZnO-fed (area under curve during the first 4 h post-feeding for milk Zn: 16.1 mg/L) and Zn-Met-fed cows (15.7 mg/L) than in control cows (15.0 mg/L). During the first 4 h post-feeding, milk to blood Zn ratio was greater in nZnO-fed animals but lower in Zn-Met-fed cows compared with control cows. Oxidative stress-related variables in plasma, blood hematological profiles, and mammary permeability related variables were not different across treatments. In summary, lactation performance, Zn concentrations in milk and plasma, hematological profiles, mammary permeability were similar in cows fed nZnO and Zn-Met. We therefore suggested that nZnO feeding can improve Zn bioavailability without impairing lactation performance, health status, and mammary gland permeability in dairy cows.

## 1 Introduction

Zinc (Zn) is an essential micronutrient for dairy cows because of its role in maintaining their health status and performance. In lipopolysaccharide-treated cows fed Zn methionine (Zn-Met) in replacement of ZnSO_4_ (40 mg/kg), somatic cell counts in milk (SCC) were reduced (Horst et al., 2019). Consequently, cows consuming diets containing recommended levels of organic/inorganic Zn were greater in milk SCC, compared with animals with insufficient Zn (66% of recommended level, in inorganic/organic form), respectively (Cope et al., 2009). However, Whitaker et al. (1997) disagreed with Cope et al. (2009). Cope et al. (2009) suggested that dairy diets with the recommended levels of 66% organic Zn led to higher milk production. In addition, other studies conducted on dairy cows with relatively lower initial milk SCC (150,000 cells/mL) found that feeding organic Zn did not affect milk SCC, but reduced numbers of developed mastitis (Spain et al., 1993). Together, these observations indicate that Zn plays an important role in maintaining the health status of the bovine mammary glands.

When dietary organic Zn is provided at 66% of recommended level (NRC 2001), early lactating cows had reduced milk production relative to animals with sufficient Zn supply (Cope et al., 2009). Moreover, when lactating cows released from heat stress were fed a Zn-Met complex, they exhibited an improved integrity of their mammary tight junctions (Weng et al., 2018). These studies indicated that in specific physiological conditions (early lactating stage or heat stress), Zn plays an important role in maintaining homeostasis and structural integrity of mammary tissue (Cope et al., 2009; Weng et al., 2018). Additionally, Wang et al. (2013) found that dietary addition of Zn-amino acid chelates and Zn-proteinate chelates not only improved milk production, but also enhanced rumen fermentation (improved total volatile fatty acids and lowered N proportion of bacteria in rumen fluid) (Wang et al., 2013). Relating to the immune system, addition of Zn-amino acid chelates and Zn-proteinate with strong chelation led to higher serum antibody titers, compared to the control (Wang et al., 2013). Another study found that addition of Zn sulfate monohydrate or Zn-Met at 500 mg/kg (dry matter (DM) basis) had no negative effects on hematological and biochemical parameters, compared to the control (Sobhanirad and Naserian, 2012). Thus, supplementing dairy cows with sufficient Zn may be beneficial for maintaining their health.

At present, nanotechnology is quickly developing, and has been applied in areas including personal care products, medicine, food packaging, and food additives (Hill and Li, 2017). Nanoparticles have properties including a larger specific surface area, greater surface activity, and high catalytic efficiency (Rajendran, 2013). The Zn oxide nanoparticles (nZnO) have recently drawn a lot of attention and have been considered to be applied potentially in the livestock industry (Wang et al., 2016). Relative to regular ZnO, dietary nZnO supplementation to the animal diets improved bioavailability, promoted animal growth rate, survival, and enhanced immunological status in weaned pigs (Pei et al., 2019). However, a safety evaluation on dietary supplementation of nZnO in lactating ruminants has not yet been conducted. By affecting oxidative stress and patterns of their mammary tight functions, oral administration of nZnO can shuffle Zn into the milk efficiently without affecting milk yield and composition in lactating mice (Cai et al., 2019). Moreover, nZnO led to amplified toxicity in MCF-7TX drug-resistant breast cancer cell lines (Ruenraroengsak et al., 2019). These studies showed potential of nZnO in feed industry of dairy cows.

To learn the effect of dietary nZnO addition on mammary metabolism and health status of lactating dairy cows, this study aimed to investigate the effects of different forms of Zn addition (nZnO and Zn-Met) on lactation performance, Zn translocation in mammary gland, hematological profiles, and variables related to oxidative stress and tight junction leakage in both milk and blood of lactating dairy cows.

## 2 Materials and methods

### 2.1 Cows and experimental design

The current study was approved by the Animal Care Committee of Zhejiang University and was conducted in collaboration with the dairy farm of Zhejiang University. Thirty multiparous Holstein cows (parity = 2.5 ± 0.20) from mid-lactating stage with days in milk 158 (standard deviation 43.2) and body weight of 694 kg (standard deviation 60.5) were selected and assigned to 10 blocks based on their parity and milk yield. They were then randomly assigned to 3 treatment groups: basal diets (control, 69.6 mg/kg DM, providing Zn at about 1,670 mg/d, NRC, 2001); basal diet with 190 mg/kg of Zn-Met (with 99% purity, CAS: 56329-42-1, providing Zn at 960 mg/d), or 50 mg/kg of nZnO (50 nm, with > 99% purity, CAS: ZH-ZnO50N, about 960 mg/d Zn). The morphology and approximate diameter of the powder form of nZnO was analyzed by scanning electron microscopy (SEM) using a HITACHI-4800S microscope (Hitachi, Japan). The size distribution spectrum of nZnO was determined using the Zetasizer Nano-ZS (Malvern Instruments, Worcestershire, UK) after the powder form nZnO was suspended in double deionized water and was dispersed (0.5 mmol/L). Cows were kept in an individual tie-stall and were given free access to water. Either Zn-Met or nZnO was fed to the animals during the morning feeding (06:00) by scattering them on total mixed rations (TMR) for each cow. Feeds were provided ad libitum to yield 10% orts. The experiment lasted for 10 wk with the first 2 wk serving as an adaptation period during which no feed additive was introduced.

### 2.2 Sampling and analysis

Feed intake was recorded for 2 consecutive days (3rd and 4th experimental days) in week 2, 4, 6, and 8, respectively. The samples of TMR and orts were collected on the same day every 2 wk throughout the study. All samples were analyzed for DM (105 °C for 5 h), crude protein (CP), method 988.05; AOAC, 1990), acid detergent fiber (ADF, method 973.18; AOAC, 1990), and neutral detergent fiber (NDF, Van Soest et al., 1991) without adding sodium sulfite and amylase. The concentrations of iodine, manganese, selenium, copper, and cobalt in the TMR were determined with inductively coupled plasma mass spectrometry (ICP-MS, PerkinElmer NexION 300X, Houston, Texas, USA, Wilschefski and Baxter, 2019). In brief, mineral trace elements were determined under the following operating conditions: cool gas flow (13 L/min), auxiliary gas flow (1.05 L/min), nebuliser gas flow (1.2 L/min), nebuliser sample uptake rate (150 μL/min). Nutrient composition of basal diet is presented in Table 1.

**Table 1:** Ingredients and compositions of the experimental diets (% as dry matter basis).

Item	Content
Ingredients	
Corn silage	37.5
Alfalfa hay	13.8
Brewer's grains	11.5
Soybean meal	10.55
Sugar beet pulp	7.59
Whole cotton seed	5.8
Oat grass	7.9
Cotton seed meal	1.91
Rapeseed meal	1.15
Sodium bicarbonate	0.59
Limestone	0.50
Premix^1^	0.40
Salt	0.27
CaHPO_4_	0.27
MgO	0.20
Met	0.07
Composition	
CP	16.9
NDF	30.5
ADF	18.8
Ash	7.02
Ether extract	5.32
NE_L_^2^, Mcal/kg DM	1.81
Zinc, mg/kg DM	69.6
Iodine, mg/kg DM	0.88
Manganese, mg/kg DM	18.75
Selenium, mg/kg DM	0.42
Copper, mg/kg DM	16.2
Ferrum, mg/kg DM	286
Cobalt, mg/kg DM	0.24

1 Formulated to provide per kilogram of dry matter diet: vitamin A 1,200,000 to 1,800,000 IU; vitamin D 280,000 to 420,000 IU; vitamin E 6,400 to 9,600 IU.

2 Net energy for lactation was calculated based on the Ministry of Agriculture of China recommendations (Feng et al., 2004).

Milk yield was recorded on the 3rd and 4th days every other week, and milk was sampled on the 4th day during week 2, 4, 6, and 8, respectively. The collected milk sample (50 mL) was mixed with bronopol (0.02 mg/mL milk, milk preservative; D & F Control Systems Inc., San Ramon, CA) and was used to analyze milk composition (fat, protein, lactose, milk urea nitrogen, and SCC) through an infrared analysis system (Laporte and Paquin, 1999) by using a 4-channel spectrophotometer (MilkoScan; Foss Electric A/S, Hillerod, Denmark). Milk Zn level was determined with ICP-MS (PerkinElmer NexION 300X, Houston, Texas, USA, Wilschefski and Baxter, 2019), on the first day of the experimental period by time points (0, 0.5, 1, 2, and 4 h relative to the morning feeding, sampling cumulative milk) and the 4th day of week 2, 4, 6, and 8. To evaluate the effects of nZnO and Zn-Met addition on mammary permeability, bovine serum albumin (BSA, Delamaire and Guinard-Flament, 2006), plasmin activity (Vries et al., 2016) in the milk was determined with a radial immunodiffusion assay (rabbit anti-BSA antiserum; Abcam, Cambridge, UK). Briefly, milk plasmin activity was determined with pyro-GLU-Phe-Lysp-nitroanilide hydroxychloride chromogenic substrate [2.5 mg/mL; Biophen CS-41(03), Aniara, Westchester, OH] in a 96-well plate (Sarstedt, Helsingborg, Sweden). To evaluate oxidative stress status in the mammary gland, milk malondialdehyde (MDA, Lin et al., 2019 was measured with a Spectra Max M5 microplate reader and commercial kit.

Blood samples (approximately 4 mL) were collected from the coccygeal vein of each cow into 5-mL tubes containing an anticoagulant (heparin lithium) at 3 h after their morning feeding on the 4th day during week 2, 4, 6, and 8, respectively. Samples were then centrifuged at 3,000 × *g* at 4 °C for 15 min to collect plasma; and the plasma was the frozen at −20 °C for subsequent analysis. On the first day of the experimental period, the whole blood was sampled from each cow at 0, 0.5, 1, 2, and 4 h relative to Zn feeding in the morning (The interval between the morning milking and afternoon milking was about 5 h. To avoid the influence of milking to the blood kinetics in the coccygeal vein, we chose to determine the general blood kinetics within 4 h). Plasma and whole blood samples were analyzed for Zn concentration by using ICP-MS (Wilschefski and Baxter, 2019), with the same operating condition referred above. Superoxide dismutase (Mohebbi-Fani et al., 2016; MB-9735B), glutathione peroxidase (Bühler et al., 2017, MB-9946B), total anti-oxidative capacity (Gu et al., 2018, MB-5729C), and MDA (Gu et al., 2018, MB-5892B) were measured by using with a Spectra Max M5 microplate reader and commercial kits. The plasma lactose concentration was determined with a commercial kit a Spectra Max M5 microplate reader (Guinard-Flament et al., 2011). On the 4th day of week 8, whole blood subsamples were collected from individual cows and were immediately placed into 10-mL tubes for later analyses of hematological indices using a hematology autoanalyzer (IDEXX Laboratories, Inc., Westbrook, Maine, USA).

### 2.3 Statistical analysis

The Zn transfer rate was defined by the following equation: Zn transfer rate = milk Zn concentration × milk yield/(dietary Zn concentration × DMI). The area under the curve (AUC) values for the Zn concentration in blood and milk collected on the first day of the experimental period by time point (0, 0.5, 1, 2, and 4 h relative to the morning feeding) were calculated using the trapezoidal rule (Cai et al., 2019). The AUC value of Zn concentrations in blood and milk, as well as hematological profiles were conducted by using ANOVA model (SAS (v. 9.4, SAS Institute Inc., Cary, NC)). All the other data were analyzed using SAS (v. 9.4, SAS Institute Inc., Cary, NC, MIXED model). For each parameter, the best covariance structure was defined with Akaike's information criterion among AR (1), unstructured, and compound symmetry, and covariance type AR (1) were used for repeated measures due to the lower AIC. A randomized complete block design with repeated measures was used for the analyses, with the week, treatment, treatment × week interaction as the main effects and with cow within diet and block as random effects. The means were separated using the PDIFF option in the LSMEANS treatment. The experimental results were reported as least squares means. Significance was declared at *P* ≤ 0.05, and 0.05 < *P* ≤ 0.10 was considered a trend.

## 3 Results

### 3.1 Lactation performance and mammary permeability

**Fig. 1:**
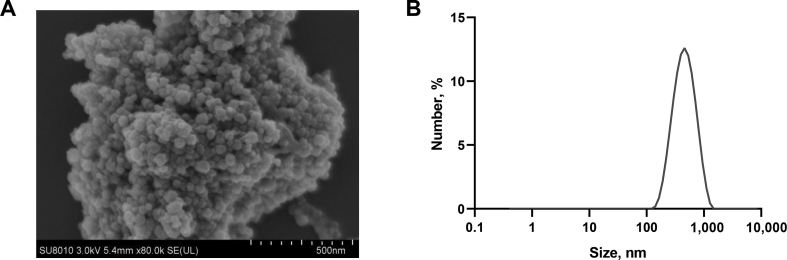
Morphology and size of nano-sized zinc-oxide. (A) Scanning electronic microscopy images. Scale bar was presented in the bottom right corner. (B) Size distributions of nano-sized ZnO in water.

**Fig. 2:**
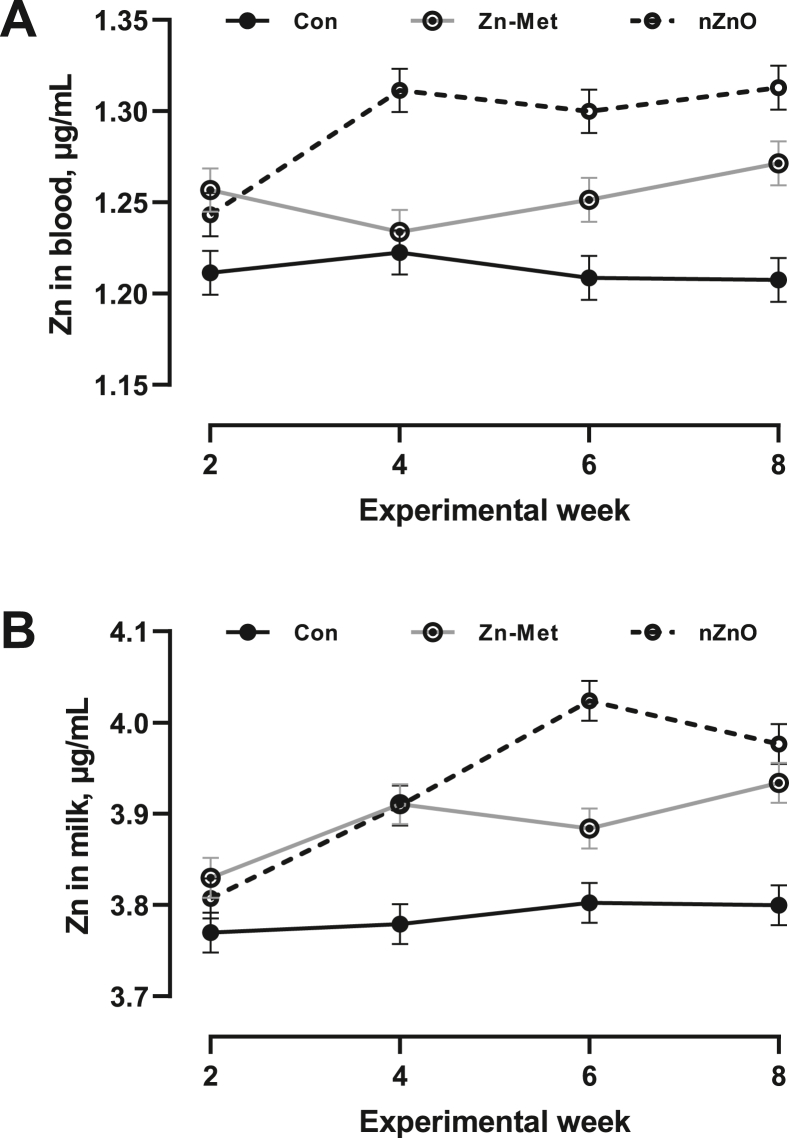
Change in zinc (Zn) concentrations in blood (A) and milk (B) of lactating cows fed basal diet without (Con) or with addition of Zn-methionine (Zn-Met) or nano-sized ZnO (nZnO).

The nZnO were observed as smooth-surfaced spheres under SEM with the expected size of 50 nm (Fig. 1A). For nZnO in water, size distribution spectra showed a larger peak compared to the expected size (Fig. 1B). The DMI (*P* = 0.58), milk yield (*P* = 0.37), and milk composition (protein (*P* = 0.35), fat (*P* = 0.70), lactose (*P* = 0.60) and milk urea nitrogen (*P* = 0.13)), as well as somatic cell score (*P* = 0.21), did not differ among cows from the 3 groups throughout the entire experimental period (Table 2). Milk Zn concentration was larger in cows fed with Zn-Met and nZnO compared to the control (*P* = 0.001, Table 2 and Fig. 2A). Moreover, Zn transfer rates were greater in cows consuming nZnO, compared to controls (*P* = 0.03). Concentrations of MDA (*P* = 0.23), BSA (*P* = 0.31), and plasmin (*P* = 0.72) in the milk were similar among 3 treatments (Table 2), respectively.

**Table 2:** Lactation performance and index related to leakage and oxidative stress in the milk of lactating dairy cows fed different zinc sources.

Item	Treatment			SEM	*P*-value		
	Control	Zinc-Met	Nano-sized zinc		Treat	Week	Treat × week
DMI, kg/d	24.3	24.1	23.6	0.43	0.58	0.53	0.71
Yield, kg/d							
Milk	32.6	32.9	33.5	0.67	0.37	<0.01	0.60
ECM^1^	36.4	38.0	38.6	1.00	0.25	0.33	0.43
Protein	1.07	1.11	1.13	0.026	0.18	<0.01	0.96
Fat	1.36	1.43	1.44	0.063	0.60	<0.01	0.32
Lactose	1.66	1.66	1.76	0.073	0.15	<0.01	0.73
Content, %	1.36	1.42	1.44	0.062	0.61	<0.01	0.30
Protein	3.29	3.40	3.29	0.067	0.35	0.02	0.31
Fat	4.22	4.38	4.16	0.196	0.70	0.11	0.68
Lactose	5.11	5.04	5.10	0.056	0.60	<0.01	0.95
MUN, mg/dL	14.3	14.9	15.2	0.41	0.13	<0.01	0.20
SCS^2^	3.87	3.80	3.43	0.213	0.21	0.07	0.86
Zinc, mg/L	3.79^b^	3.89^a^	3.93^a^	0.025	<0.01	<0.01	0.04
Zinc transfer rate^3^, %	4.75^b^	4.89^ab^	5.24^a^	0.119	0.03	<0.01	0.31
Leakage index							
BSA, g/L	0.58	0.62	0.61	0.021	0.31	0.71	0.86
Plasmin, U/L	1.85	1.82	1.87	0.040	0.72	0.87	0.40
MDA, mmol/L	45.4	41.9	42.4	1.54	0.23	0.85	0.71

DMI = dry matter intake; ECM = energy-corrected milk; MUN = milk urea nitrogen; SCS = somatic cell score; MDA = malondialdehyde; BSA = bovine serum albumin.

^a, b^ Within a row, values with a different superscript suggested a statistical difference across the different treatment groups.

1 ECM (kg) = 0.3246 × milk yield (kg) + 13.86 × milk fat (kg) + 7.04 × milk protein (kg) (Orth, 1992).

2 SCS = log_2_ [somatic cell counts (10^3^/mL)/100] + 3.

3 Zinc transfer rate = milk zinc concentration × milk yield/(dietary zinc concentration × DMI).

### 3.2 Concentrations and appearance of Zn in milk and plasma

Plasma Zn concentration was higher in cows fed Zn-Met and nZnO than the control cows (*P* = 0.001, Table 3 and Fig. 2B), with higher levels was observed in nZnO-fed cows than in Zn-Met cows (*P* = 0.003). The plasma concentration of oxidative variables including MDA (*P* = 0.75), superoxide dismutase (*P* = 0.40), glutathione peroxidase (*P* = 0.62), and total anti-oxidative capacity (*P* = 0.39), as well as lactose (*P* = 0.35), were similar among 3 treatment groups, respectively. Moreover, all hematological variables were not different across all the treatments (Table 4).

**Table 3:** Effects of different zinc sources on plasma variables in lactating dairy cows.

Item	Treatment			SEM	*P*-value		
	Control	Zinc-Met	Nano-sized zinc		Treat	Week	Treat × week
Zinc, mg/L	1.21^c^	1.25^b^	1.29^a^	0.012	<0.01	0.44	0.31
MDA, nmol/mL	2.66	2.57	2.66	0.096	0.75	0.83	0.66
SOD, U/mL	107.3	107.6	111.9	2.71	0.40	0.75	0.45
GSH-Px, U/mL	128.6	124.6	125.9	3.05	0.62	0.10	0.88
T-AOC, U/mL	3.76	3.88	3.98	0.115	0.39	0.60	0.84
Lactose, μmol/L	113.4	113.2	109.9	1.93	0.35	0.41	0.78

MDA = malondialdehyde; SOD = superoxide dismutase; GSH-Px = glutathione peroxidase; T-AOC = total antioxidant capacity.

^a, b, c^ Within a row, values with a different superscript suggested a statistical difference across the different treatment groups.

**Table 4:** Effects of different addition of zinc sources on the hematological profiles of lactating dairy cows.

Item	Control	Zinc-Met	Nano-sized zinc	SEM	*P*-value
RBC, × 10^6^/μL	6.33	6.05	5.89	0.158	0.17
Reticulocyte, × 10^9^/L	1.09	1.39	0.94	0.28	0.52
MCV, fL	48.8	49.3	47.1	0.93	0.25
MCH, pg	17.1	17.4	16.9	0.29	0.37
MCHC, g/dL	35.1	35.3	35.8	0.27	0.16
RDW, fL	26.7	26.2	26.9	0.72	0.79
Haemoglobin, g/dL	10.7	10.3	9.9	0.26	0.14
WBC, × 10^6^/μL	11.9	12.2	12.2	0.89	0.77
Neutrophils, × 10^9^/L	5.23	4.37	4.65	0.42	0.36
Lymphocytes, × 10^9^/L	3.90	4.51	4.66	0.91	0.83
Monocytes, × 10^9^/L	2.50	2.92	2.49	0.36	0.64
Eosinophils, × 10^9^/L	0.26	0.38	0.32	0.05	0.33
Basophils, × 10^9^/L	0.024	0.025	0.030	0.009	0.87
Neutrophils, %	45.6	39.6	40.5	3.12	0.37
Lymphocytes, %	30.9	34.2	36.1	3.11	0.50
Monocytes, %	21.0	22.0	20.4	2.2	0.88
Eosinophils, %	2.29	3.97	2.85	0.75	0.30
Basophils, %	0.24	0.24	0.27	0.09	0.96
HCT, %	30.3	29.1	28.0	0.81	0.16
Platelets, × 10^3^/μL	332	326	383	24.5	0.25
MPV, fL	10.14	10.37	9.84	0.19	0.16
PDW, %	7.09	7.17	6.78	0.19	0.35
Procalcitonin, %	0.34	0.34	0.38	0.03	0.49

RBC = red blood cells; MCV = mean corpuscular volume; MCH = mean corpuscular hemoglobin; MCHC = mean corpuscular hemoglobin concentration; RDW = red blood cell distribution width; WBC = white blood cells; HCT = hematocrit; MPV = mean platelet volume; PDW = platelet distribution width.

**Fig. 3:**
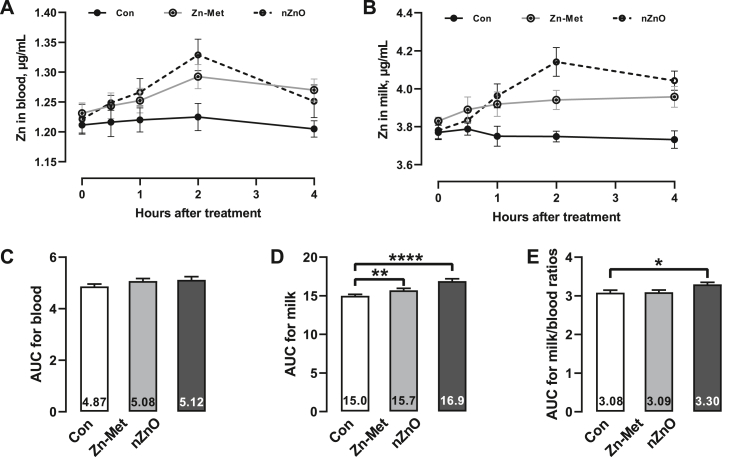
Zinc (Zn) concentrations in the blood and milk of lactating cows fed basal diet without (Con) or with addition of Zn-methionine (Zn-Met) or nano-sized ZnO (nZnO). (A) Change in blood Zn concentration. (B) Change in milk Zn concentration. (C, D and E) Area under the curve (AUC) during the first 4 h post-feeding for blood, milk, and milk to blood ratios. ∗*P* < 0.05, ∗∗*P* < 0.01, and ∗∗∗∗*P* < 0.0001. The bars indicate the SEM.

Effects of Zn appearance in plasma and milk within 4 h after feeding are shown in Fig. 3. Cows fed nZnO (2 h, *P* = 0.007) and Zn-Met (2 h, *P* = 0.03; and 4 h, *P* = 0.03) had higher plasma Zn concentrations than control cows (Fig. 3A). Milk Zn concentrations were higher (*P* < 0.05) in nZnO- (1 h, *P* = 0.004; 2 h, *P* = 0.001; and 4 h, *P* = 0.001) and Zn-Met-fed cows (1 h, *P* = 0.02; 2 h, *P* = 0.009, and 4 h, *P* = 0.003) than in control cows (Fig. 3B), with higher levels observed in nZnO-fed animals than in Zn-Met-fed cows in 2 h (*P* = 0.006). The AUC for Zn in the blood did not differ across all the groups (Fig. 3C, *P* = 0.49). However, cows fed nZnO and Zn-Met had higher AUC for milk Zn than control cows (Fig. 3D, *P* = 0.004). The AUC for the milk to blood ratio was higher in nZnO-fed cows than in controls (Fig. 3E, *P* = 0.04).

## 4 Discussion

Previously, the effects of Zn addition on the lactation in dairy cows was investigated (Cope et al., 2009; Wang et al., 2013). Cope et al. (2009) suggested that lactating cows fed TMR containing recommended Zn (in organic form) levels had greater milk yield than cows fed diets containing 66% of recommended Zn. Moreover, Wang et al. (2013) found that addition of Zn-proteinate chelate (strong chelation), but not Zn-proteinate chelate (moderate chelation) nor Zn sulfate, improved the milk yield of cows in early lactation compared to the control. Sobhanirad et al. (2010) found that dietary addition of Zn sulfate monohydrate or Zn-Met had no positive effect on milk yield, relative to the control-cows. In our present study, lactation performance was similar across animals fed basal, Zn-Met added, and nZnO added diets, suggesting that neither Zn-Met nor nZnO altered milk production. The discrepancy could be attributed to lactation stage and Zn availability. Relating to the findings of Cope et al. (2009), all cows evaluated in our current study were fed a basal diet containing a Zn level that well meets the cow requirements according to NRC (2001). Our study suggested that when cows were supplied with sufficient Zn from the basal diet, Zn-Met or nZnO addition only altered Zn concentrations in the blood and milk, but not their milk production and composition. The limited response of milk yield and composition in cows fed nZnO and Zn-Met could be compromised by Zn source in the basal diet. The Zn is an essential trace element, and plays important roles in immune system, development, reproduction and wound healing (Salas-Huetos et al., 2017; Chan et al., 2005). Daily Zn intake needs to be guaranteed for human health. It is recommended that adults should consume Zn at a level of 11 mg/d, and should be lower than 100 mg/d to avoid potential health risk (Chan et al., 2005). The recommend daily milk consumption is 732 mL/d (Mullie et al., 2016), providing Zn at a level of 2.9 mg/d (30% of recommended dose) under the current experimental condition. However, it is still worth learning if Zn would be over-accumulated when Zn in the premix is solely from nZnO.

Desai et al. (1996) found that metals with particle sizes lower than 100 nm can be absorbed more quickly compared to metals with bigger particle sizes. Smaller particle sizes can enhance the bioavailability of metal particles after absorption from intestinal regions (Qiu et al., 2019). Thus, the higher Zn content in the plasma of nZnO-fed cows than in that of Zn-Met-fed cows suggests that the bioavailability of nZnO is higher than that of Zn-Met; however, similar Zn concentrations in the milk of nZnO- and Zn-Met-fed cows suggested that the proportion of Zn transferred into milk was similar. Although cows fed nZnO and Zn-Met were similar in terms of milk Zn concentrations, Zn from nZnO seemed to entered into milk more rapidly, driven by the higher Zn concentrations in the blood, compared with cows in Zn-Met group. Additionally, plasma Zn in the form of nZnO was transferred into milk more rapidly than Zn-Met. The AUC for the milk to blood ratio and Zn transfer rate suggested that a larger proportion of Zn was transferred from feed into milk in nZnO-fed cows than in the control-cows. The higher proportion of Zn transferred from blood to milk in nZnO-fed cows may be attributed to their greater Zn concentrations in the blood. The mechanism of how nZnO is transported into the mammary gland still remains to be investigated.

A wide range of studies has been conducted to clarify the effects of nZnO on blood immune cells in vitro. The toxicity of nZnO during reactive oxygen species generation, autophagic cell death, or cell apoptosis has been brought up (Tripathy et al., 2014; Johnson et al., 2015). However, in our recent in vivo study, we observed that most of nZnO particles were actually degraded in the mammary gland of lactating mice exposed to oral nZnO, and did not cause any dysfunction of the mammary gland (Cai et al., 2019). In our present study, cows fed with nZnO showed no changes in blood hematological profiles, compared with cows fed basal diet and Zn-Met. These observations indicated that feeding nZnO did not cause toxic effects to the blood circulation of dairy cows. Moreover, nanoparticles seemed to be inducers of cellular oxidative stress both in vitro (Duan et al., 2016; Tee et al., 2016) and in vivo studies (Abbasalipourkabir et al., 2015). Specifically, ZnO nanoparticles undergo spontaneous redox cycling because of their inherent defects during manufacture and reactive oxygen species triggering trait (Persaud et al., 2020). To assess the potential risks of ZnO, we further determined plasma oxidative stress variables from cows in the 3 groups, the results suggested that feeding nZnO did not affect oxidative stress. Similarly, mammary gland tight junction maintenance provides the structural basis of high milk production in dairy cows, and concentrations of plasmin and BSA in milk and plasma lactose levels were considered as indices reflecting mammary tight junction integrity (Wang et al., 2019). Limited changes in these parameters across the 3 groups of cows suggested that nZnO addition did not affect tight junction integrity.

## 5 Conclusion

Compared with Zn-Met, nZnO feeding improved Zn concentrations in the blood and transfer rate of feed into milk in mid-lactating dairy cows, without exerting any negative effects on milk yield and composition, as well as their health status, including oxidative stress status, and mammary tight junction integrity. Thus, nZnO may potentially serve as an alternative zinc source with high bioavailability.

## Author contributions

**Jie Cai:** Investigation, Data curation, Software, Writing – original draft. **Chao Miao:** Investigation, Data curation, Software. **Yi Chen:** Investigation. **Yunyi Xie:** Investigation. **Jianxin Liu:** Conceptualization, Writing – review & editing. **Diming Wang:** Conceptualization, Methodology, Supervision, Writing – review & editing.

## Conflict of interest

We declare that we have no financial and personal relationships with other people or organizations that can inappropriately influence our work, and there is no professional or other personal interest of any nature or kind in any product, service and/or company that could be construed as influencing the content of this paper.
